# Potential of Microalgae Extracts for Food and Feed Supplementation—A Promising Source of Antioxidant and Anti-Inflammatory Compounds

**DOI:** 10.3390/life12111901

**Published:** 2022-11-16

**Authors:** Fernando Pagels, Helena M. Amaro, Tânia G. Tavares, Berta F. Amil, A. Catarina Guedes

**Affiliations:** 1 CIIMAR—Interdisciplinary Centre of Marine and Environmental Research, University of Porto, Novo Edifício do Terminal de Cruzeiros de Leixões, Avenida General Norton de Matos, 4450-208 Matosinhos, Portugal; 2 FCUP—Faculty of Science, University of Porto, Rua do Campo Alegre, 4169-007 Porto, Portugal; 3 LEPABE—Laboratory for Process Engineering, Environment, Biotechnology and Energy, Faculty of Engineering, University of Porto, Rua Dr. Roberto Frias, 4200-465 Porto, Portugal; 4 LAQV/REQUIMTE—Faculty of Pharmacy, University of Porto, Rua de Jorge Viterbo Ferreira, 228, 4050-313 Porto, Portugal

**Keywords:** *Isochrysis*, *Nannochloropsis*, *Phaeodactylum*, *Tetraselmis*, ethanolic extract, water extract, polysaccharides

## Abstract

Microalgae are known producers of antioxidant and anti-inflammatory compounds, making them natural alternatives to be used as food and feed functional ingredients. This study aimed to valorise biomass and exploit new applications and commercial value for four commercially available microalgae: *Isochrysis galbana*, *Nannochloropsis* sp., *Tetraselmis* sp., and *Phaeodactylum tricornutum*. For that, five extracts were obtained: acetone (A), ethanol (E), water (W), ethanol:water (EW). The antioxidant capacity (ABTS^•+^/DPPH^•^/^•^NO/O_2_^•−^/ORAC-FL) and anti-inflammatory capacity (HBRC/COX-2) of the extracts were screened. The general biochemical composition (carbohydrates, soluble proteins, and lipids) and the main groups of bioactive compounds (carotenoids, phenolic compounds, and peptides) of extracts were quantified. The results of antioxidant assays revealed the potential of some microalgae extracts: in ABTS^•+^, *Nannochloropsis* sp. E and *Tetraselmis* sp. A, E, and P; in DPPH^•^, *Tetraselmis* sp. A and E; in ^•^NO, *P. tricornutum* E and EW; in O_2_^•−^, *Tetraselmis* sp. W; and in ORAC-FL, *I. galbana* EW and *P. tricornutum* EW. Concerning anti-inflammatory capacity, *P. tricornutum* EW and *Tetraselmis* sp. W showed a promising HBRC protective effect and COX-2 inhibition. Hence, *Tetraselmis* sp. and *P. tricornutum* extracts seem to have potential to be incorporated as feed and food functional ingredients and preservatives.

## 1. Introduction

Microalgae comprise a large and diverse group of photosynthetic microorganisms. As producers of secondary metabolites such as pigments, fatty acids, polyphenols, and peptides, these organisms can be a great source of natural products. Nowadays, several species are grown on a large scale and are used as nutritional supplements due to the presence of bioactive compounds, such as β-carotene, astaxanthin, and polyunsaturated fatty acids (PUFAs) [1].

These organisms form the basis of the marine food chain and play an important nutritional role for marine species. Hence, among many industrial applications of microalgal biomass, aquaculture feed is included, in which microalgae are essential feed sources for crustaceans, molluscs, and fish larvae and juveniles, as well as throughout the life cycle of bivalve molluscs [1,2].

The use of microalgae biomass and algae-derived products for feed is a prospective path for expanding the animal production sector in a more sustainable and environmentally friendly way that is an alternative to traditional terrestrial agriculture [3]. Species such as *Isochrysis galbana*, *Nannochloropsis* sp., *Tetraselmis* sp., and *Phaeodactylum tricornutum*, among others, have been pointed out as good sources of proteins, carbohydrates, and lipids to be used as feed ingredients, either for aqua feed or animal feed [4]. Additionally, in feed formulations, besides its nutritional value, the incorporation of low levels of microalgal biomass has been shown to aid the growth of fish larvae, as well as increase the survival rates, the animal ingestion, and the pigmentation of aquaculture and poultry products. Finally, microalgae have also been associated with an enhancement of the swim bladder function of fish [4]. Additionally, feed enriched with microalgae has been shown to enhance animal physiology, improving the immune response, disease resistance, and gastrointestinal function, as well as antiviral and antibacterial protection [3]. However, the presence of cellulosic cell walls of some eukaryotic microalgae species can induce some digestibility problems. This was observed either in microalgae fish/shrimp feed formulations, but also in humans when using *Tetraselmis* sp. and *P. tricornutum* as food ingredients [5]. Moreover, the human consumption of animal meat fed with microalgae enhances the food’s nutritional value and content of bioactive compounds [4].

Following the above-mentioned, microalgae have been used as a human food source or nutritional supplements for hundreds of years, as a register of the use of spirulina (*Arthrospira* spp.) during the Aztec Empire proves [6]. On the contrary, the current consumption of microalgae as a food ingredient is usually only related to nutraceuticals and “healthier” foods. Nutraceuticals are supplements derived from food or food products that enrich the diet and aid in the prevention and treatment of diseases and disorders [7]. Furthermore, there is a growing interest in natural antioxidants as replacements for synthetic compounds due to the increased safety concerns. Many synthetic antioxidants (e.g., butylated hydroxyanisole (BHA), butylated hydroxytoluene (BHT)) used as food preservatives are considered to have carcinogenic and/or toxic effects on animal models [8]. Thus, algal antioxidant compounds have great potential for improving the oxidative stability of food products [9].

Furthermore, the positive effects of microalgal compounds on the prevention of oxidative degeneration and inflammation, the reduction of cardiovascular and degenerative diseases, as well as the control and treatment of certain tumours is evidenced in several studies (reviewed elsewhere [10,11]). The main identified compounds with benefits for human health are pigments, polysaccharides, and PUFAs. However, other compounds, such as peptides and polyphenols, have been studied in more detail in recent years [10]. 

Additionally, from a biotechnological standpoint, microalgae are a diverse group of organisms with enormous potential that has yet to be fully explored, and only a few species are produced on a large basis. *Chlorella* and spirulina (*Arthorspira*) are the most produced species worldwide, but *Phaeodactylum tricornutum*, *Tetraselmis* sp., and *Isochrysis galbana* are also among the most produced in Europe [12]. In general terms, the main applications of microalgae biomass are cosmetics (24%), food supplements and nutraceuticals (24%), and feed (19%). Concomitantly, a significant effort is currently being made in research and development to generate more feed and nutraceutical products from microalgae biomass. The development of extracts and their direct application in functional food/feed is one of the most often used procedures. The cost of extracting and processing products from these extracts is somehow justified once the higher bioactive potential and applicability are higher than the raw biomass [13]. Moreover, the value market of microalgal extracts can increase to around 40-fold when compared to raw biomass [12]. To obtain a microalgal extract, solvent selection is fundamental, considering the compound’s solubility, toxicity, final use, and environmental impact of residues. Aiming for a more sustainable process, efforts were taken to ascertain the influence of food GRAS (Generally Recognized as Safe) solvents upon the recovery of bioactive extracts [14].

Thus, in this study, food-grade extracts of four commercially available microalgae biomass, namely, *I. galbana*, *Nannochloropsis* sp., *Tetraselmis* sp., and *P. tricornutum*, were screened and studied aiming biomass valorisation, exploiting their commercial value as bioactive food supplements with antioxidant and anti-inflammatory features. 

## 2. Materials and Methods

### 2.1. Microalgae Biomass Source

*Isochrysis galbana*, *Nannochloropsis* sp., *Tetraselmis* sp., and *Phaeodactylum tricornutum* dry biomass (Phytobloom^®^) was kindly provided by Necton (Olhão, Portugal).

### 2.2. Extraction Procedure

For each of the selected microalgae, four different extracts were obtained with GRAS solvents: acetone (A), ethanol (E), water (W), and a mixture of ethanol:water (1:1, *v*/*v*) (EW). The extractions were performed with a Precellys homogenizer (Bertin, France), in triplicate, using 250 mg of dry biomass in 3 cycles with 5 mL of solvent each (6 series of 8000 rpm for 30 s with 45 s of pause). Extracts were centrifuged at 2000× *g* for 10 min, and the supernatant was dried—A and E by a rotavapor system, and W by a rotary evaporator; EW extracts were dried using both systems.

A fifth extract was obtained using water with further precipitation with cold ethanol, in a 1:3 ratio for the obtaining of polysaccharide-rich extracts (P) [15]. The solution was centrifuged at 2000× *g* for 10 min, and the supernatant was dried in a drying oven (60 °C).

All extracts were saturated with nitrogen to avoid oxidation and stored in low humidity (desiccator), in the dark, until further analyses.

### 2.3. Biochemical Characterization

Extracts were characterized in terms of soluble proteins, total lipids, and total carbohydrates. The content of proteins, lipids, and carbohydrates was determined in triplicate and expressed as a percentage of extract weight (%).

Soluble protein content was quantified using bovine serum albumin as standard and phosphate buffer as solvent by bicinchoninic acid (BCA) based on the PierceTM BCA Protein Assay Kit (Thermo Scientific, Rockford, IL, USA).

The content of lipids was quantified gravimetrically by a chloroform/methanol (2:1, *v*/*v*) extraction [16] using 100 mg of extract in 5 mL of solvent. Then the nonpolar fraction was collected, dried, and weighed to determine the lipid content of the extract.

Carbohydrate content was quantified spectrophotometrically through the phenol sulfuric acid method, using glucose as standard and water as solvent [17].

### 2.4. Antioxidant Capacity Assessment

The antioxidant capacity of each extract was accessed by ABTS^•+^ [18], DPPH^•^ [19], O_2_^•−^ [20], and ^•^NO [20] scavenging assays and the oxygen radical absorbance capacity (ORAC-FL) assay [21]. All the assays were performed in triplicate, and extracts were resuspended in 20% DMSO at a concentration of 4 mg mL^−1^ and further diluted for each assay. Trolox was used as a positive control and gold standard to validate and quantify the antioxidant capacity of extracts through the establishment of a calibration curve. However, to compare the antioxidant capacity of the extracts, their concentration able to scavenge 50% of the radicals (IC_50_) was calculated with GraphPad Prism software (version 8.0) through curve spline interpolation, while the ORAC value was calculated from the Trolox equivalent and expressed as mg_TE_ g^−1^. 

### 2.5. Anti-Inflammatory Capacity Assessment

#### 2.5.1. Human Red Blood Cell (HRBC) Membrane Stabilization by Heat-Induction

The HRBC assay was performed according to [22]. Fresh blood was collected intravenously from a healthy human volunteer into heparinized tubes to prevent coagulation. Blood was centrifuged at 1250× *g* for 5 min and washed three times with an equal volume of sodium phosphate buffer (PBS, 10 mM, pH 7.4). HRBC was reconstituted at a 40% (*v*/*v*) suspension with isotonic PBS. Salicylic acid (3.6 mM) was used as a positive control, and 20% DMSO (in PBS) as the negative control. Extracts were tested at a concentration of 500 μg mL^−1^. Inhibition of HRBC degradation was calculated for each extract and expressed as a percentage of inhibition (%).

#### 2.5.2. Cyclooxygenase (COX-2) Enzymatic Activity

Inhibition of COX-2 enzymatic activity was performed in the extracts with a positive result in the HRBC stabilization assay. A COX inhibitor screening assay kit (Cayman Chemical) was used according to the manufacturer’s instructions, and PGF2α was used as a positive control. Dried extracts were resuspended in DMSO and tested at a concentration of 500 μg mL^−1^.

### 2.6. Bioactive Compounds

Specific groups of known bioactive compounds (total carotenoids, total peptides, and total phenolic compounds) were identified and quantified spectrophotometrically in the bioactive extracts to find connections with the found antioxidant and anti-inflammatory bioactivities. Hence, in resuspended extracts in acetone, total carotenoids were quantified according to the equation A_480_ × 4.0 [23]. Total phenolic compounds (TPC) content was determined by the Folin–Ciocalteu method [24] in resuspended extract in methanol:water (4:1, *v*/*v*), and TPC were quantified using gallic acid as standard. Total peptides were quantified using a 3 kDa membrane to filtrate the extract and further quantification through the BCA protein assay, as described before.

### 2.7. Statistical Analysis

Experimental data were analysed using GraphPad Prism V. 8.0. A Shapiro–Wilk test of normality was performed before the analysis of variance (ANOVA) to confirm the normal distribution of the residuals. A two-way ANOVA with Tukey’s multi-comparison test was used to find differences between microalgae and solvents in the antioxidant capacity, anti-inflammatory capacity, and biochemical composition between extracts.

## 3. Results

### 3.1. Extraction Yield

The feasibility of an extract for a commercial application depends on its potential and extraction yield, as an industry depends on a marketable product. The extraction rate is directly related to the solvent used and the species targeted. Table 1 shows the yield of the studied extracts. Overall, extraction using water led to a high yield in all species. On the other hand, *Phaedoactylum* spp. polysaccharide extracts attained the lowest yield (0.40 ± 0.01%_DW_), which indicates that it might be not economically viable in large-scale processing.

### 3.2. Biochemical Composition

The biochemical profiles of all the produced extracts were evaluated to better understand the potential of the screened microalgae (Table 2). The biochemical contents of the extracts, albeit with some differences between species, followed similar patterns. In terms of soluble proteins, W and P extracts (ca. 10%_Extract_) contained the highest content in all four species, while in terms of carbohydrates, the polysaccharide-rich extract (P) had the highest content, as expected (>70%_Extract_). In terms of lipids, E and A extracts had the highest content, with values varying from 50 to 72%_DE_, depending on the species.

### 3.3. Antioxidant Capacity

**Figure 1:**
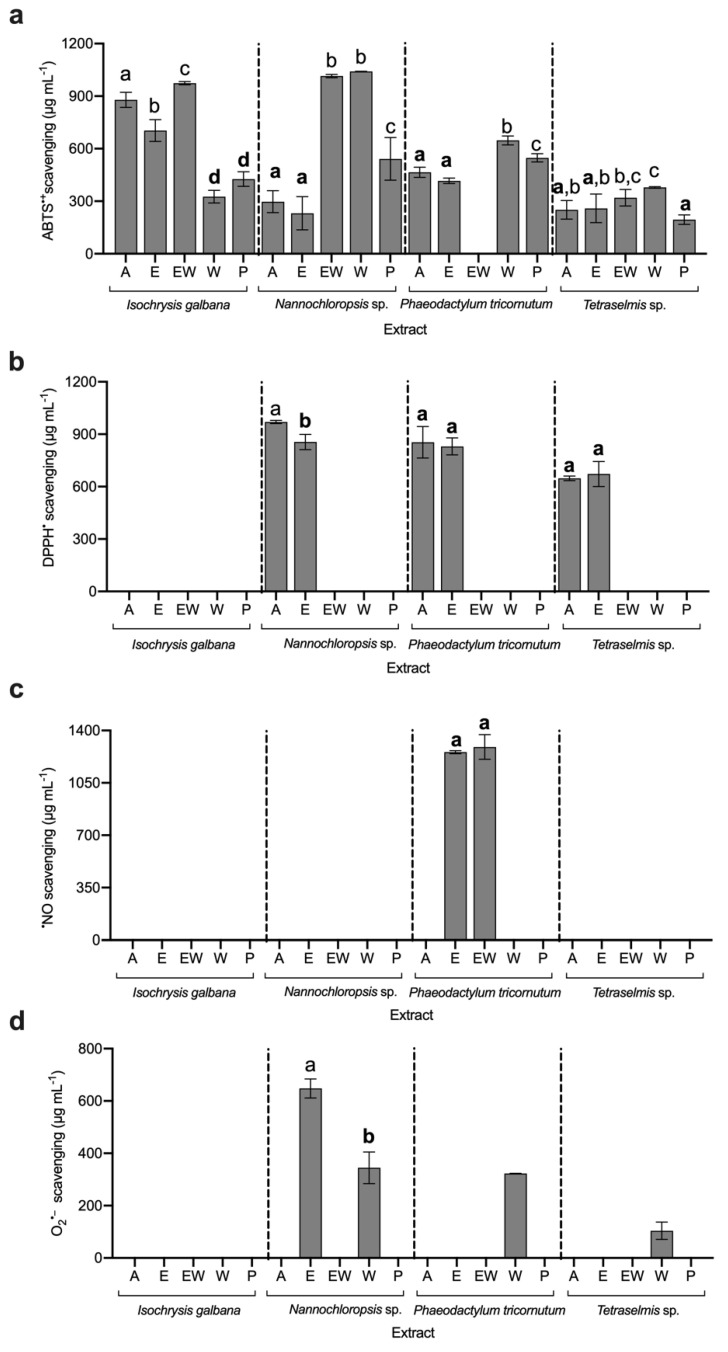
IC_50_ of microalgae extracts for (**a**) ABTS^•+^, (**b**) DPPH^•^, (**c**) ^•^NO, and (**d**) O_2_^•−^ scavenging assays (average ± standard deviation, n = 3). Extracts: acetone (A), ethanol (E), ethanol:water (EW), water (W), and polysaccharides (P). Different letters above bars for each alga indicate statistical differences (*p* > 0.05). The bold letters indicate the lowest IC value per species.

**Figure 2:**
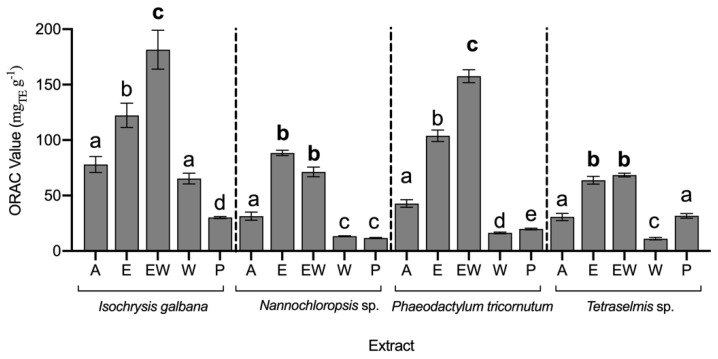
ORAC value (mg_TE_ g^−1^) of microalgae extracts (average ± standard deviation, n = 3). Extracts: acetone (A), ethanol (E), ethanol:water (EW), water (W), and polysaccharides (P). Different letters above bars for each alga indicate statistical differences (*p* > 0.05). The bold letters indicate the lowest IC value per species.

The evaluation of antioxidant capacity is critical for the valorisation of microalgal biomass; however, due to the complex antioxidant system of these organisms, the evaluation must consider a variety of components. The antioxidant capacity of microalgal extracts was determined using five different approaches allowing for a better understanding of these organisms’ bioactive potentials (Figure 1 and Figure 2).

Concerning general radical scavenging, evaluated by the ABTS^•+^ assay (Figure 1a), it was possible to ascertain 19 IC_50_ from the 20 extracts, and the lowest value found was an average of 246.7 μg mL^−1^, with no statistical differences (*p* > 0.05), for *Nannochloropsis* sp. E extract, and for *Tetraselmis* sp. A, E, and P extracts. For the DPPH^•^ assay (Figure 1b), it was only possible to calculate the IC_50_ for six extracts, and the best values were found in *Tetraselmis* sp. A and E extracts, with an average of 659.9 μg mL^−1^.

In the case of specific radicals, for the ^•^NO assay (Figure 1c), only for two extracts was it possible to determine the IC_50_ of *P*. *tricornutum* E and EW, with a concentration of 1255.3 ± 10.0 and 1290.0 ± 82.8 μg mL^−1^, respectively. For the O_2_^•−^ assay (Figure 1d), the IC_50_ was found for four extracts; the lowest IC_50_ value was found to be for *Tetraselmis* sp. W extract, with a concentration of 104.1 ± 33.1 μg mL^−1^.

Regarding the ORAC-FL assay (Figure 2), although all extracts appeared to have an antioxidant effect, E and EW extracts seemed to have higher potential, regardless of the species. Contrary to IC_50_ assays, the highest ORAC-FL values were those with the highest antioxidant capacity. The highest values were found in *I. galbana* EW and *P. tricornutum* EW, at 181.6 ± 17.5 and 157.7 ± 5.9 mg_TE_ g^−1^, respectively.

### 3.4. Anti-Inflammatory Capacity

**Figure 3:**
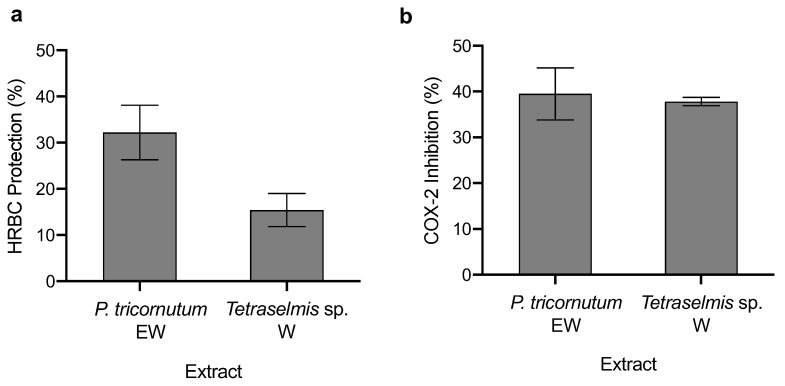
The anti-inflammatory capacity of microalgae extracts in terms of (**a**) protection of HRBC thermal degradation and (**b**) inhibition of COX-2 enzyme (average ± standard deviation, n = 3). Extracts: *P. tricornutum*, EW (ethanol:water), *Tetraselmis* sp. W (water).

Although all extracts were evaluated in terms of HRBC protection against thermal degradation, only two extracts had protective effects (Figure 3), namely, *P. tricornutum* EW and *Tetraselmis* sp. W extracts, *P. tricornutum* EW being the one with the highest protective effect (32.2 ± 5.90%), twice the effect of *Tetraselmis* sp. W extract. Therefore, for the COX-2 inhibition assay, only these two extracts were evaluated, both attaining an inhibition of ca. 38% of the enzymatic activity in a concentration of 500 μg mL^−1^.

### 3.5. Bioactive Potential of Microalgae Extracts

The bioactive potential of microalgae is related to the ability of these organisms to produce several types of natural compounds. Some compounds such as pigments (specially carotenoids), phenolic compounds, and peptides are usually associated with antioxidant and anti-inflammatory capacity. Therefore, for each of the evaluated assays, the total content of these groups was determined in the best extracts. Table 3 shows the total carotenoid, phenolic compound, and peptide contents for the most promising extracts, taking the seven evaluated assays.

In terms of carotenoids, the highest content was found in *Nannochloropsis* sp. E (14.8 ± 0.3 mg g_Extract_^−1^). As expected, nonpolar solvents, such as ethanol and acetone, could extract nonpolar compounds such as carotenoids compared to more polar solvents such as ethanol:water. Hence, in W and P extracts, carotenoids were not quantified.

Regarding phenolic compounds, and due to their diverse chemical structure, no specific trend was observed regarding their extractability according to different solvents. Nonetheless, the highest content was found in *I. galbana* EW and *Tetraselmis* sp. A and E extracts.

Concerning the content of peptides, *I. galbana* EW contained the highest amount of peptides, 1.6-fold higher than the extract of *P. tricornutum* EW.

## 4. Discussion

As stated before, microalgal products hold great potential for antioxidant and anti-inflammatory applications in functional feed and nutraceutical supplementation [25]. Antioxidants can reduce or eliminate reactive oxygen species (ROS). In microalgae cells, they can be enzymatic or nonenzymatic in nature and can be produced intracellularly or extracellularly. The main known mechanism of action of antioxidant compounds are singlet O_2_ quencher, radical scavenger, electron donor, hydrogen donor, peroxide decomposer, enzyme inhibitor, gene expression regulation, and metal-chelating agents [26]. Because of the great variety of compound structures and mechanisms of action, as well as the diversity of ROS, it is critical to accurately measure the antioxidant capacity of algae extracts. Microalgal compounds such as pigments, peptides, and polyphenols have been described as capable of scavenging different types of radicals [13].

In this study, all the evaluated extracts seem to have a potential antioxidant capacity to some extent. Considering all the evaluated assays, the best extracts are *I. galbana* EW, *Nannochloropsis* sp. E, *P. tricornutum* E and EW, and *Tetraselmis* sp. A, E, W, and P. Regarding the correlation between specific assays and a group of compounds, it is noteworthy that those with the highest potential in general scavenging assays (ABTS^•+^ and DPPH^•^) are obtained with nonpolar solvents, usually related to pigments and phenolic compounds, while more specific radicals (^•^NO and O_2_^•−^) are better scavenged by polar solvents (E, EW, and P). In the ORAC-FL assay, the antioxidant capacity is usually related to bioactive peptides, which accords with the content found in *I. galbana* EW and *P. tricornutum* EW extracts.

*I. galbana* is mainly associated with the aquaculture industry due to its high lipid content that can reach up to 30%DW, being a considerable source of ω3 PUFAs [27]. Moreover, the known high content of polysaccharides in this microalga has been described as a source of antioxidant and antimicrobial components [28]. This potential was also confirmed in this study, with the highest yield of extraction in P extracts from the four species and a high antioxidant capacity with the EW extract, which may have extracted both groups of compounds. Although the carotenoid profile has not been evaluated in this study, Gilbert-López et al. [29] evaluated the commercial biomass of *I. galbana*. The authors observed a high concentration of fucoxanthin, it being the main carotenoid present in the microalga.

When it comes to *Nannochloropsis* sp., this microalga is also mainly exploited in aquaculture due to its high content of PUFAs, carotenoids, polyphenols, and vitamins [30]. In the present study, the ethanolic extract seems to have potential in terms of antioxidant capacity, besides being mainly composed of lipids (>60%_Extract_). Additionally, from the eight best extracts, *Nannochloropsis* sp. E was shown to contain the highest amount of carotenoids (15 mg g_Extract_ ^−1^). The lipid profile within *Nannochloropsis* spp. produced in several conditions was reviewed by Zanella and Vianello [30], and it was observed that only eicosapentaenoic acid (EPA, ω3) represented up to 10% of total lipids and 3% of the biomass. In the case of carotenoids, xanthophylls such as zeaxanthin, antheraxanthin, violaxanthin, and lutein represent ca. 50% of carotenoids. These two groups of compounds have high biotechnological interest due to their bioactive capacities and relevant biological roles. Those features can explain the high antioxidant capacity found in this study, indicating the potential of valorisation of this extract, particularly to be incorporated into nutraceutical products.

Regarding *P. tricornutum*, this microalga is a marine diatom and has gained great interest in research due to the high amount of PUFAs, especially ω3 fatty acids [31]. Moreover, oil containing EPA from *P. tricornutum* is one of the few products approved by the European Union for human consumption of vitamins [30]. Apart from PUFAs, *P. tricornutum* can be also a source of fucoxanthin, as it is the main carotenoid found in this microalga [32]. From *P. tricornutum* extracts, E and EW were selected with high antioxidant potential. Both extracts contain carotenoids and lipids and have similar contents of phenolic compounds. Rico et al. [33] identified myricetin and catechin as the predominant phenolic compounds in *P. tricornutum*, which are both known for their antioxidant potential; moreover, it seems that the profile can change within growth conditions and strains, which may change the final potential of the biomass.

Moreover, *Tetraselmis* sp. is a marine green microalga widely used in aquaculture as live feed for bivalve molluscs, crustaceans, and rotifers, mainly due to its high protein content and antioxidant compounds [34]. Furthermore, *Tetraselmis chui* is already approved by the European Union for human consumption and has been proposed as an excellent source of vitamin C and E and PUFAS (ω3 and ω6) vitamins [30,34]. From the evaluated extracts, four extracts of *Tetraselmis* sp. have shown potential antioxidant capacity (A, E, W, and P). *Tetraselmis* sp. nonpolar extracts (A and E) seem to have a high content of phenolic compounds, while W and P extracts seem to have peptides and possibly bioactive polysaccharides, as 74% of P extract is composed of carbohydrates. A similar polysaccharides extract was obtained by Kashif et al. [35] and showed antioxidant and antifungal potential.

Furthermore, anti-inflammatory capacity is one of the important biological features observed in different metabolites from microalgae such as *Chlorella, Dunaliella,* and *Phaeodactylum,* among others. The highest anti-inflammatory capacity is usually related to polysaccharides, polyunsaturated fatty acids (PUFAs), and carotenoids [13,36]. Inflammation is the immune system’s natural physiological response to tissue injury, which occurs as the organism deals with viruses, harmful chemicals, and injured cells. These stimuli can generate acute or chronic inflammatory reactions in several organs, tissue damage, and immune-mediated illnesses. As a result, it is critical to find alternatives that minimize inflammation while avoiding or exacerbating the body’s immune reaction [36]. Interestingly, the extracts selected in this study as anti-inflammatory were also highlighted as the best in ^•^NO, O_2_^•−^ antioxidant assays, which are ROS usually associated with the inflammatory process.

The anti-inflammatory capacity of *P. tricornutum* extracts was also observed by Neumann et al. [32], where ethanolic and water extracts reduced the pro-inflammatory cytokines in human blood mononuclear cells and murine macrophages. The anti-inflammatory capacity of these extracts was associated with the presence of fucoxanthin, EPA in the ethanolic extract, and sulphated polysaccharides in water extracts. Here, *P. tricornutum* EW was the only *P. tricornutum* extract to show a protective effect on HBRC, and consequently, the only one evaluated in terms of COX-2 inhibition. *P. tricornutum* EW has a similar amount of lipids and carbohydrates in the extract (ca. 20%), which may indicate that the mixture of solvents could have extracted EPA, fucoxanthin, and polysaccharides. From the quantified groups of bioactive compounds, *P. tricornutum* EW showed a low content of carotenoids, but a relatively high amount of total phenolic compounds and peptides, which may also be part of this anti-inflammatory capacity.

On the other hand, *Tetraselmis* sp. water extract showed a lower HBRC protective effect but a similar COX-2 inhibition compared to *P. tricornutum* EW.

Additionally, the anti-inflammatory capacity of *Tetraselmis* spp. extracts have been suggested by Jo et al. [37], where 80% methanol extract exhibited the strongest anti-inflammatory effect in LPS-stimulated RAW 264.7 cells, reducing the production of NO and pro-inflammatory cytokines.

Overall, A, E, and W extracts from *Tetraselmis* sp. and EW from *P. tricornutum* seem to be the most promising due to their antioxidant and anti-inflammatory capacities.

The total antioxidant activity of *P. tricornutum* EW and *Tetraselmis* sp. W obtained by ORAC is in the same magnitude as that obtained in methanolic extracts of *Nannochloropsis* granulate, *Neochloris oleoabundans*, and *Scenedesmus obliquus* (Banskota et al. 2019). Furthermore, in terms of the specific antioxidant activity of *P. tricornutum* EW and *Tetraselmis* sp. W, the obtained IC_50_ for O_2_^•−^ was higher than those reported before for *Gloethece* sp. ethanolic, acetonic, and lipidic extracts, and water extracts of *Cyanobium* sp. (Costa et al. 2020, Pagels 2020, Amaro et al., 2021). However, lower ^•^NO values were obtained in comparison to ethanolic and acetonic extracts of *Gloeothece* sp. and *Cyanobium* sp. water extracts (Pagels 2020, Amaro et al., 2021).

In terms of Cox-2 activity of *P. tricornutum* EW and *Tetraselmis* sp. W extracts, the obtained results (ca. 38%) are higher than those registered as *Tetraselmis* mutants (6–30%) and *Skeletonema* sp. (17%) water extracts, but similar to *Cyanobium* sp. aqueous extracts (Pagels et al. 2021, Cardoso 2019). In terms of the HRBC results of *P. tricornutum* EW (ca. 32%) and *Tetraselmis* sp. W (ca. 16%), they are lower than those observed in a *Gloeothece* sp. hexane:isopropanol 3:2 (*v*/*v*) extract (61.6 ± 9.2) and a *Thalassiosira weissflogii* methanolic extract (ca. 79%), but similar to an ethyl extract of *Gloeothece* sp. (ca. 14.8) (Amaro 2022; Shalini). However, it should be highlighted that the *P. tricornutum* and *Tetraselmis* sp. EW and W extracts have an advantage in terms of the extraction process, as they use food grade, low-cost solvents with low environmental impacts compared to the other lipids extracts, which are critical requirements for up-scaling (Pagels 2021).

**Figure 4:**
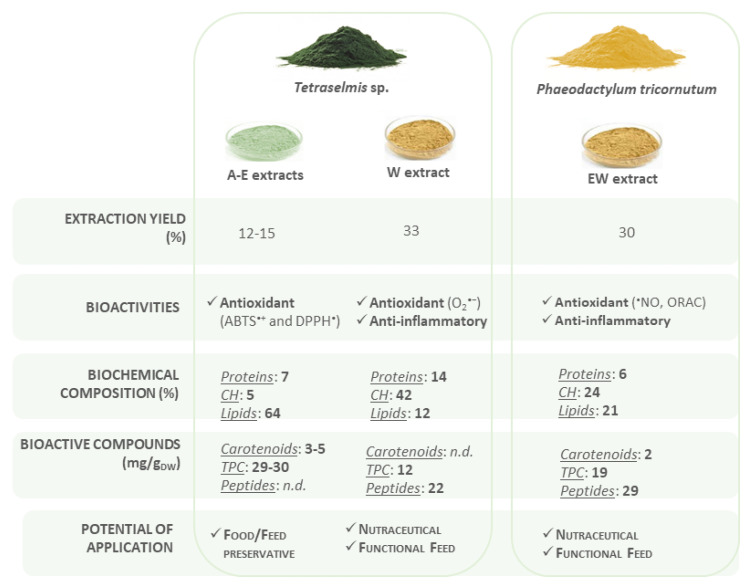
Schematic representation of the overall potential of microalgae extracts using GRAS solvents. CH: carbohydrates; TPC: total phenolic compounds.

Such extracts have been demonstrated to have the potential to be incorporated into supplements for feed and food, the full prospective of which is depicted in Figure 4.

Additionally, due to the different natures of the extracted compounds of *Tetraselmis* sp. (E and W), the extraction of several bioactive compounds from this species can be considered in the future using a biorefinery concept, as explored before by Assunção et al. [38]. This could support the more sustainable and profitable exploitation of *Tetraselmis* by obtaining two potential bioactive products from the same biomass, with yield extraction of up to 48%. Additionally, the use of E and W as food ingredient extracts could increase the sensorial acceptance as a food ingredient when compared to whole microalga biomass while keeping the bioactive potential [39]. Additionally, the inclusion of bioactive microalgal extracts instead of biomass has the advantage of increasing digestibility due to the absence of microalga cellulose-rich cell walls. This was observed either in microalgae fish/shrimp feed formulations or using *Tetraselmis* sp. and *P. tricornutum* as food ingredients [5,40].

Furthermore, the remaining biomass can be used for other purposes, for which the production of biofuels and biofertilizers may be included, increasing the sustainability and economic feasibility of all processes [41].

## 5. Conclusions

The potential of four commercially available microalgae biomass was screened to be a source of antioxidant and anti-inflammatory compounds, envisioning a food or feed ingredients application. Hence, only the use of GRAS solvents was considered to extract several kinds of bioactive compounds produced by these organisms. In vitro antioxidant and anti-inflammatory screening assays revealed that eight extracts may have a potential bioactive application: *I. galbana* EW, *Nannochloropsis* sp. E, *P. tricornutum* E and EW, and *Tetraselmis* sp. A, E, and W. Particularly, *Tetraselmis* sp. and *P. tricornutum* extracts seem to be the most promising ones to be further exploited due to both their antioxidant and anti-inflammatory features.

## Figures and Tables

**Table 1:** Extraction yield (average ± standard deviation, n = 3) of microalgae biomass using different solvents. Extracts: acetone (A), ethanol (E), ethanol:water (EW), water (W), and polysaccharides (P). Bold letters represent the highest value for each species. The different lower-case letters indicate statistical differences between extracts of the same species (*p* < 0.05).

Microalgae	Extract	Yield (%_DW_)
*Isochrysis galbana*	A	19.46 ± 0.62 ^a^
	E	25.44 ± 0.76 ^b^
	EW	33.84 ± 0.88 ^c^
	W	**67.27 ± 1.24 ^d^**
	P	21.47 ± 0.85 ^e^
*Nannochloropsis* sp.	A	9.56 ± 1.02 ^a^
	E	10.40 ± 0.23 ^a^
	EW	18.57 ± 0.02 ^b^
	W	**26.07 ± 1.36 ^c^**
	P	6.48 ± 0.44 ^d^
*Phaeodactylum tricornutum*	A	12.12 ± 0.67 ^a^
	E	15.67 ± 0.97 ^b^
	EW	**29.90 ± 1.36 ^c^**
	W	**32.09 ± 1.03 ^c^**
	P	0.40 ± 0.01 ^d^
*Tetraselmis* sp.	A	12.03 ± 2.80 ^a^
	E	14.94 ± 0.94 ^a^
	EW	21.96 ± 2.07 ^b^
	W	**33.21 ± 1.28 ^c^**
	P	13.59 ± 0.61 ^a^

**Table 2:** Biochemical composition of selected microalgae extracts in terms of soluble protein, carbohydrate, and lipid contents (average ± standard deviation, n = 3). Extracts: acetone (A), ethanol (E), ethanol:water (EW), water (W), and polysaccharides (P). Bold letters represent the highest value for each species. The different lower-case letters indicate statistical differences between extracts of the same species (*p* < 0.05).

Microalgae	Extract	Content (%_Extract_)		
		Soluble Proteins	Carbohydrates	Lipids
*Isochrysis galbana*	A	4.47 ± 0.04 ^a^	0.80 ± 0.05 ^a^	51.36 ± 1.46 ^a^
	E	**9.90 ± 0.31 ^b^**	2.39 ± 0.08 ^b^	**63.11 ± 2.28 ^b^**
	EW	6.79 ± 0.47 ^c^	20.97 ± 0.34 ^c^	25.67 ± 0.60 ^c^
	W	**9.65 ± 1.18 ^b^**	53.26 ± 7.78 ^d^	13.77 ± 0.28 ^d^
	P	**9.65 ± 0.17 ^b^**	**79.40 ± 0.28 ^e^**	2.65 ± 1.17 ^e^
*Nannochloropsis* sp.	A	3.59 ± 0.17 ^a^	2.41 ± 0.07 ^a^	**71.82 ± 1.36 ^a^**
	E	6.99 ± 0.27 ^b^	3.49 ± 0.08 ^b^	**71.12 ± 3.12 ^a^**
	EW	6.26 ± 0.80 ^b^	24.48 ± 0.54 ^c^	45.66 ± 5.35 ^b^
	W	8.80 ± 0.96 ^c^	50.80 ± 2.46 ^d^	36.29 ± 2.57 ^b^
	P	**11.10 ± 0.32 ^d^**	**77.51 ± 3.12 ^e^**	8.33 ± 3.61 ^c^
*Phaeodactylum tricornutum*	A	2.94 ± 0.41 ^a^	1.75 ± 0.05 ^a^	**66.74 ± 3.79 ^a^**
	E	5.01 ± 0.61 ^b^	3.79 ± 0.11 ^b^	**68.11 ± 0.53 ^a^**
	EW	6.47 ± 0.44 ^c^	23.45 ± 0.44 ^c^	20.61 ± 1.93 ^b^
	W	**11.38 ± 1.39 ^d^**	38.95 ± 4.26 ^d^	26.86 ± 0.55 ^c^
	P	**13.60 ± 1.25 ^d^**	**73.19 ± 5.56 ^e^**	0.00 ± 0.00 ^d^
*Tetraselmis* sp.	A	3.99 ± 0.30 ^a^	3.16 ± 0.05 ^a^	**69.24 ± 3.09 ^a^**
	E	6.90 ± 0.38 ^b^	5.05 ± 0.07 ^b^	**64.11 ± 2.66 ^a^**
	EW	6.28 ± 0.68 ^b^	21.49 ± 0.23 ^c^	44.65 ± 1.69 ^b^
	W	**13.78 ± 0.66 ^c^**	41.82 ± 4.31 ^d^	11.57 ± 2.27 ^c^
	P	12.00 ± 0.53 ^d^	**74.04 ± 5.51 ^e^**	6.78 ± 1.55 ^c^

**Table 3:** Bioactive compound contents (mg g_Extract_^−1^) of selected antioxidant and anti-inflammatory extracts (mg g_Extract_^−1^, average ± standard deviation, n = 3). Extracts: acetone (A), ethanol (E), ethanol:water (EW), water (W), and polysaccharides (P). Different letters for each bioactive compound group indicate statistical differences between extracts (*p* > 0.05).

Microalgae	Extract	Highlighted Potential (Assay)	Carotenoids	Phenolic Compounds	Peptides
*Isochrysis galbana*	EW	ORAC-FL	2.6 ± 0.2 ^a^	**35.5 ± 1.6 ^a^**	**50.4 ± 5.3 ^a^**
*Nannochloropsis* sp.	E	ABTS^•+^	**14.8 ± 0.3 ^b^**	16.0 ± 0.6 ^b^	-
*Phaeodactylum tricornutum*	E	^•^NO	7.9 ± 0.2 ^c^	22.8 ± 1.2 ^c^	-
	EW	^•^NO, ORAC-FL, HBRC, COX	2.0 ± 0.1 ^d^	19.0 ± 0.7 ^d^	29.6 ± 3.2 ^b^
*Tetraselmis* sp.	A	ABTS^•+^, DPPH^•^	5.4 ± 0.2 ^e^	28.5 ± 1.6 ^e^	-
	E	ABTS^•+^, DPPH^•^	3.4 ± 0.2 ^f^	30.7 ± 1.1 ^e^	-
	W	O_2_^•−^, HBRC, COX	-	12.5 ± 0.1 ^f^	22.4 ± 0.5 ^c^
	P	ABTS^•+^	-	8.8 ± 0.2 ^g^	13.4 ± 1.0 ^d^

## Data Availability

Not applicable.
